# Expanded catalogue of metagenome-assembled genomes reveals resistome characteristics and athletic performance-associated microbes in horse

**DOI:** 10.1186/s40168-022-01448-z

**Published:** 2023-01-12

**Authors:** Cunyuan Li, Xiaoyue Li, Rongjun Guo, Wei Ni, Kaiping Liu, Zhuang Liu, Jihong Dai, Yueren Xu, Shamshidin Abduriyim, Zhuangyuan Wu, Yaqi Zeng, Bingbing Lei, Yunfeng Zhang, Yue Wang, Weibin Zeng, Qiang Zhang, Chuangfu Chen, Jun Qiao, Chen Liu, Shengwei Hu

**Affiliations:** 1 College of Life Science, Shihezi University, Shihezi, 832003 Xinjiang China; 2 Key Laboratory of Ecological Corps for Oasis City and Mountain Basin System, Shihezi University, Shihezi, 832003 Xinjiang China; 3 College of Animal Science and Technology, Shihezi University, Shihezi, 832003 Xinjiang China; 4 Novogene Bioinformatics Institute, Beijing, 100000 China; 5 State Key Laboratory of Sheep Genetic Improvement and Healthy Production, Xinjiang Academy of Agricultural and Reclamation Science, Shihezi, 830003 Xinjiang China; 6 Xinjiang Altay Animal Husbandry and Veterinary Station, Altay, 836501 Xinjiang China; 7 College of Animal Science, Xinjiang Agricultural University, Urumqi, 830000 Xinjiang China

**Keywords:** Athletic performance, Antimicrobial resistance gene, Gut, Horse, Metagenome-assembled genome, Nanopore sequencing

## Abstract

**Background:**

As a domesticated species vital to humans, horses are raised worldwide as a source of mechanical energy for sports, leisure, food production, and transportation. The gut microbiota plays an important role in the health, diseases, athletic performance, and behaviour of horses.

**Results:**

Here, using approximately 2.2 Tb of metagenomic sequencing data from gut samples from 242 horses, including 110 samples from the caecum and 132 samples from the rectum (faeces), we assembled 4142 microbial metagenome-assembled genomes (MAG), 4015 (96.93%) of which appear to correspond to new species. From long-read data, we successfully assembled 13 circular whole-chromosome bacterial genomes representing novel species. The MAG contained over 313,568 predicted carbohydrate-active enzymes (CAZy), over 59.77% of which had low similarity match in CAZy public databases. High abundance and diversity of antibiotic resistance genes (ARG) were identified in the MAG, likely showing the wide use of antibiotics in the management of horse. The abundances of at least 36 MAG (e.g. MAG belonging to *Lachnospiraceae*, *Oscillospiraceae*, and *Ruminococcus*) were higher in racehorses than in nonracehorses. These MAG enriched in racehorses contained every gene in a major pathway for producing acetate and butyrate by fibre fermentation, presenting potential for greater amount of short-chain fatty acids available to fuel athletic performance.

**Conclusion:**

Overall, we assembled 4142 MAG from short- and long-read sequence data in the horse gut. Our dataset represents an exhaustive microbial genome catalogue for the horse gut microbiome and provides a valuable resource for discovery of performance-enhancing microbes and studies of horse gut microbiome.

Video Abstract

**Supplementary Information:**

The online version contains supplementary material available at 10.1186/s40168-022-01448-z.

## Background

Horses (*Equus caballus*) are used worldwide in leisure, racing, transportation, agriculture, and forestry activities, and they are widely raised for the production of milk and animal protein in developing countries [1]. There are an estimated 59 million horses worldwide [2], with an annual economic impact of approximately $300 billion [3]. Thus, it is important to achieve a detailed understanding of horse biology to potentially improve the use of horse in human activities.

Horses have been employed as a model system for investigating digestion in herbivores with hindgut fermentation, which include donkey, zebra, rhinoceros, and elephant, and for studying ruminant caecal digestion [4, 5]. The intestinal tract of horses contains a large number of symbiotic bacteria, fungi, archaea, protozoa, and virus [6], which provide animals with the hydrolytic enzymes that convert carbohydrates into energy by fibre fermentation [7, 8]. The metabolites produced from these microbial groups in horses play important roles in host health, diseases, development, and even behaviour [9–15]. Prior studies have reported that the intestinal microbiota is associated with horse diseases (e.g., colitis, laminitis, grass sickness, asthmatic, diarrhoea) [16, 17] and exercise [18, 19]. Mach et al. used blood transcriptome, blood metabolome, and faecal microbiome to study horses’ endurance before and after races and proposed that the gut-mitochondrial axis was associated with athletic performance [15]. Thus, an in-depth understanding of the microbial composition of the horse intestine offers opportunities for enhancing athletic performance and improving the maintenance of animal health by manipulating the microbiota through dietary intervention or the addition of probiotics.

The microbiota composition in horse gut has been partly characterized based mainly on 16S amplicon sequencing [9–15], Recently, a gene catalogue and hundreds of MAG of horse gut microbiome have been reported [6, 20, 21], but large-scale studies on microbiota composition and function in horses are still lacking. The emergence of high-throughput sequencing and metagenome binning technology has made it possible to obtain nearly complete metagenome-assembled genomes (MAG) on a large scale [22]. Metagenomic sequencing technology can identify a large number of previously unknown bacterial species among intestinal microbes and has been used to characterize the functions of these microbes at the genomic level [22–26]. This technology has generated thousands of MAG from humans [23], ruminants [24], chickens [25], pigs [26], and horse [6]. Moreover, long-read sequencing can improve the quality of assembly by increasing the continuity of genome assembly [27]. Thus, the use of high-throughput sequencing technology combined with long-read sequencing offers a promising strategy for characterizing the microbial composition and function of the horse microbiome. Moreover, performance-enhancing microbes and novel enzymes (e.g. CAZy) in the horse gut may be deeply explored by the whole-genome metagenomics [27].

In this study, we aimed to develop a microbial genome resource for research on the horse gut microbiome and to use that resource to answer questions about how the gut microbiota is related to racehorse performance. We used gut samples of 242 horses from two provinces of China. A large-scale metagenomic sequencing scan was performed to characterize microbiota composition in horse gut. Furthermore, we revealed resistome characteristics and athletic performance-associated microbes in horses. This study provides an exhaustive catalogue of MAG in horse and answers important questions about the relationship between the gut microbiome and horse performance.

## Methods

### Metagenomic samples

All details about experimental horses and samples are listed in Additional file 1: Table S1. A total of 242 gut samples from indigenous Yili horses, Thoroughbred horses, Tibetan horses, Shetland ponies, and Yili racehorses were collected and used in this study. These horses were raised in different counties of two provinces in China. Indigenous Yili horses (*n* = 110, Yili horse) and Tibetan horses (*n* = 10, Tibet horse) grazed on natural pastures and consumed pasture forage. Thoroughbred horses (*n* = 58, Thoroughbred) belonged to stabled horses and were fed a mixed forage and concentrate diet. Shetland ponies (*n* = 8, Shetland pony) were raised in a farm and fed a mix of pasture grasses and concentrate diet. Yili racehorses (*n* = 21, Yili racehorse) consumed a mix of pasture grasses supplemented with concentrate feedstuffs. These Yili racehorses were enrolled and winner in the 30-km category in the horse race of the Chinese nation. Faeces of racehorses were collected before the race. Through consulting the racehorse owner, we knew that these racehorses had different levels of training every day. A group of age-matched Yili nonracehorse (*n* = 35, Yili nonracehorse) was fed a mix of pasture grasses supplemented with concentrate feedstuffs and used as control group for Yili racehorses. All Yili nonracehorse have no training plan. All horses in this study were healthy and did not receive any antimicrobial treatment (antibiotic, anthelmintics, or anti-inflammatory nonsteroidal treatments) for two months before sampling. For 242 gut samples in this study, 110 caecal samples were collected from 110 indigenous Yili horses, and the remaining 132 rectal gut (faeces) samples were from 132 horses including Tibet horse, Thoroughbred, Yili racehorse, and Yili nonracehorse. All samples were frozen in liquid nitrogen, transported to the laboratory, and preserved in a freezer at −80°C until DNA extraction.

### DNA extraction and quality control

To obtain high-quality microbial DNA from the gut contents, a modified hexadecyltrimethylammonium bromide (CTAB) method was used for DNA extraction [28]. Briefly, 1000 μl of CTAB lysis buffer (0.1 M Tris-HCl [pH 8.0], 1.4 M NaCl, 0.02 M EDTA, 2% CTAB, DNA- and RNA-free) was added to a 2.0-ml EP tube (DNase- and RNase-free), along with 20 μl of lysozyme (DNA- and RNA-free); a 100-mg sample was then added, and the lysis solution was incubated for 2–3 h at 65°C. For complete lysis, the sample was mixed by inversion several times during incubation. After brief centrifugation, 950 μl of the supernatant was transferred to a new 2.0-ml EP tube containing 950 μl of a phenol–chloroform-isoamyl alcohol (25:24:1, pH 8.0, DNA- and RNA-free) solution. After mixing, the sample was centrifuged at 12,000 rpm for 10 min. The supernatant was carefully transferred to a new EP tube, and an equal volume of chloroform-isoamyl alcohol (24:1, DNA- and RNA-free) was added, followed by thorough mixing and centrifugation at 12,000 rpm for 10 min. After centrifugation, the supernatant was transferred to a new 1.5-ml EP tube, a 3/4 volume of isopropanol was added to the supernatant, and the tube was placed at −20°C for 20 min to allow precipitation after mixing. The tube was then centrifuged at 12,000 rpm for 10 min, the supernatant was discarded, and the precipitate was washed twice with 1 ml of 75% ethanol. After drying, 50 μl of ddH_2_O was added to dissolve the DNA sample. Finally, 1 μl of RNase A was added, and the sample was placed at 37°C for 15 min to digest the RNA. The quality of the extracted DNA was checked via 1% agarose gel electrophoresis, and the integrity and potential contamination of the sample were assessed via pulsed-field gel electrophoresis. For purity determination and precise quantification, a Nanodrop Kit (Implen, CA, USA) and a Qubit® 2.0 fluorometer (Life Technologies, CA, USA) were used, respectively.

### Library construction and sequencing

To prepare the Illumina sequencing library, we used the NEBNext®Ultra™ DNA Library Preparation Kit (New England Biolabs, USA) and a 1 μg DNA sample. An index code was added to the primer to allow different samples to be distinguished in the sequence data. The kit manufacturer’s recommendations were strictly followed. Briefly, the extracted DNA was fragmented into 350-bp fragments via sonication, and the ends were repaired. An adenine nucleotide was then added at the ends, and full-length adaptor sequences were connected. These libraries were purified with the AMPure XP system (Beckman Coulter, Brea, CA, USA). An Agilent 2100 Bioanalyzer and real-time PCR were used for size distribution and quantitative analyses of the purified products. After the quality of the library was verified, all of the samples were subjected to paired-end sequencing by using the Illumina NovaSeq 6000 platform with a read length of 150 base pairs (PE150).

For the preparation of the PromethION library, we used an SQK-LSK109 Ligation Sequencing Kit (Oxford Nanopore Technologies, Oxford, UK) to obtain 8 μg of DNA from each sample according to the manufacturer’s recommendations. Briefly, the DNA was processed by using a Megaruptor (Diagenode, NJ, USA); DNA fragments longer than 10 kb were then screened by using BluePippin. A specific barcode was added to the fragments that had been repaired, an A tail was also added, and the length of the fragments was checked. To complete the preparation of the DNA library, samples with different barcodes were mixed in equimolar amounts and purified, and the DNA concentration was quantified by using a Qubit fluorometer. After the quality of the library was verified, nanopore sequencing technology was used for sequencing.

### Data quality control

For the quality control of the raw data obtained from the Illumina sequencing platform, Readfq ver. 8.0 software was used to filter out low-quality reads with ambiguous “N” bases (More than 10 consecutive N bases) and adapter contamination (the overlap of the adapter sequence exceeds 15bp) with the parameters ‘--Q 53,40 --C 53,40 --N 10 --alen 15 --amis 3 --dup (--amis: cut-off adapter mis-match bases ; --dup: filter duplications)’ [29]. Briefly, the quality value filter value is set to Q20. Alignment was performed with Bowtie 2 software (version 2.2.4, http://bowtie-bio.sourceforge.net/bowtie2/index.shtml) with the parameters ‘--end-to-end, --sensitive, -I 200, -X 400’ [30], and reads aligned to the horse genome (EquCab3.0: GCA_002863925.1) were removed. The remaining high-quality reads were further analysed.

For the Nanopore sequencing data, we used Guppy software base calling to convert the data from fast5 format into fastq format. NanoPlot ver. 1.18.2 (https://github.com/wdecoster/NanoPlot/) was used to perform quality control on the fastq-format data; the threshold was set to Q > 7, and the parameters were set to ‘-t 20, --loglength, --N50’ [31]. In addition, we used BLASTR ver. 5.1 software with the parameters ‘--nproc 30, --bestn 10, --nCandidates 10, --noSplitSubreads, --maxScore -1000, --maxLCPLength 16’ to compare our data with the horse genome to filter out reads that might have originated from the horse host [32]. Reads longer than 500 bp constituted the final valid dataset.

### Metagenomic assembly and binning

SOAPdenovo2 ver. 2.04 software was used with the parameters ‘-d 1, -M 3, -R, -u, -F, -K 55’ [33] to assemble clean data from the Illumina sequencing results and to obtain scaffolds. The assembled scaffolds were then interrupted at the N junction to obtain scaftigs, and fragments shorter than 500 bp were simultaneously filtered out to obtain high-quality scaftigs [30]. MetaWRAP ver. 1.2.1 software (https://github.com/bxlab/metaWRAP) was subsequently used for metagenomic binning. In brief, the assembly was binned with the metaWRAP binning module by using the metagenomic binning programs MaxBin2, metaBAT2, and CONCOCT. The metaWRAPBin refinement module was applied to consolidate multiple binning predictions into a new, improved bin set [34]. All of the final bins were aggregated, and dRep ver. 1.1.2 software was then used with the parameters ‘-p 16, -comp 80, -con 10, -str 100, -strW 0’ [27] to remove duplicate bins. Then, dRep was used with the secondary clustering at the threshold of 99% ANI with at least 25% overlap between genomes. CheckM software ver. 1.0.7 was used to evaluate the quality of the assembled bins, which were screened according to the criteria of completeness ≥50% and contamination ≤10% (4142 medium-quality MAG) [35, 36]. Only bins assessed by CheckM as complete ≥80% and contamination ≤10% were further screened as high-quality MAG. For duplicate bins, bin scores were given as completeness − 5 × contamination + 0.5 × log(genome N50); the MAG with the highest score was retained. After analysis, 2272 MAG were retained in the assembled data (2240 MAG were assembled from the Illumina sequence data and 32 from the Nanopore sequence data). The metawrap quant_bins module was used with options ‘metawrap quant_bins -b genomes/ -o QUANT_BINS/’ to calculate the abundance of MAG in each sample [34].

Flye ver 2.4.2 2 software was used to assemble clean data from the Nanopore sequencing results with the parameters ‘--threads 4, -- meta, -g 5m’ [37]. Finally, the Illumina sequencing data were used to correct the Nanopore data, and the preprocessed clean data were compared to the scaftig data to obtain unused PE reads with the parameters ‘--end-to-end, --sensitive, -I 200, -X 400’. After scaftigs less than 2 Mb long were filtered out [38], the assembled scaftigs were subjected to statistical analysis.

Finally, PhyloPhlAn ver. 3.0.51 software [39] was used to construct a phylogenetic tree of the 4142 assembled MAG. RNAmmer ver. 1.2 software [40] was used to predict the 16S rRNA genes, and tRNAscan-SE ver1.3.1 [41] was used to predict tRNA genes. The ANI was calculated by using PYANI ver. 0.2.10 with the parameters ‘-m, ANIb’ [42].

### Gene catalogue construction, taxonomic annotation, and abundance profiling

MetaGeneMark (prokaryotic GeneMark.hmm ver. 2.10; http://topaz.gatech.edu/GeneMark/) [43] was used to predict all open reading frames (ORFs) of the assembled scaftigs (≥ 500 bp). Then, a nonredundant gene catalogue was then constructed by using CD-HIT ver. V4.5.8 software (http://www.bioinformatics.org/cd-hit) with greater than 95% identity over 90% of the shorter ORF length clustered together by a greedy pairwise comparison implemented. The longest ORF from each group was selected as the representative of the group. CD-HIT with default parameters during analysis except ‘-G 0, -aS 0.9, -g 1, -d 0, -c 0.95 b -n 5’ [44]. The uniqueness of our gene catalogue was assessed by clustering with the reported equine gut gene catalogue using the same parameters. To determine the abundance of genes and reads, these sequences were mapped to the gene catalogue (unigenes) by using Bowtie ver. 2.2.4 with the parameters ‘--end-to-end, --sensitive, -I 200, -X 400’. The genes with ≤2 reads in each sample were filtered out, and the gene catalogue was ultimately used for subsequent analysis [45]. The abundance of genes was calculated by counting the number of reads and normalizing result according to gene length.

The unigenes were aligned to the integrated NR database (2018-01-02) by using DIAMOND ver. 0.9.9.110 with the parameters ‘-k 50, -sensitive, -e 1e-5’ [46]. For the final aligned results of each gene, significant matches were defined according to an *e*-value ≤ 10×*e*-value of the top hit, and the taxonomic level was determined by using the lowest common ancestor-based algorithm, implemented in MEGAN [47]. The abundance of a taxonomic group in each sample was equal to the sum of the abundance of genes annotated to a feature [48].

### Metagenomic assignment

ORFs were predicted from the assembled MAG by using MetaGeneMark (prokaryotic GeneMark.hmm, ver. 2.10) [49]. All of the predicted genes were aligned with the integrated NR database by using DIAMOND ver. 0.9.9.110 (https://github.com/bbuchfink/diamond). In addition, GTDB-Tk (v.1.3.0) was used to assign the taxonomy of the MAG [50].

### Proteome analysis

DIAMOND software was used to search the predicted amino acid sequences of each MAG against the UniProt TrEMBL database (ver. 2020-05, https://www.uniprot.org/statistics/TrEMBL) [51] with the parameter ‘blastp’ according to an *e*-value ≤1*e*−5. From the BLAST results for each sequence, the best BLAST hit was selected for subsequent analysis [52]. Sequences that could not be aligned were defined as unknown proteins. To understand the functions of the assembled MAG, we used DIAMOND software to compare the predicted proteins to the KEGG database (ver. 2018-01-01, http://www.kegg.jp/kegg/) to obtain KEGG orthologues to determine the functional pathways in which the MAG participated. All of the predicted proteins were searched against the CAZy database (ver. 201801, http://www.cazy.org/), using dbCAN2 [53] and HMMER (version 3.3.1) [54] to annotate CAZy. Based on these results, we counted the number of functional genes with nonzero abundances [29]. PUL were predicted for Bacteroidetes MAG by using PULpy software [27]. The sex and breed were regarded as environmental factors and used Mantel test analysis in the vegan package of R 4.0.4 to test the correlation between the cazy abundance matrix and the environmental factor matrix with default parameters [55]. Resistance Gene Identifier (RGI, version 5.1.0) software was used to align the predicted proteomes to CARD (version 3.0.8) with the parameter setting blastp [56, 57].

### Faecal RNA extraction and real-time RT–PCR

Faecal total RNA was extracted from frozen tissues after grinding under liquid nitrogen using TRIzol (Invitrogen, CA, USA) according to the manufacturer’s protocol. The quantity and purity of total RNAs were detected by using NanoDrop 2000 Spectrophotometer (Thermo Scientific, Wilmington, DE). The isolated RNA was employed to synthesize cDNA using an RT–PCR kit (Takara, Dalian, China). The RT Primer Mix (Mixture of Random 6 mers and Oligo dT Primer) included in the kit was used as the primer for RT–PCR. All real-time RT–PCR analyses were performed using TB Green (TaKaRa Biotech, Dalian) according to the manufacturer’s protocol. All primer information is listed in Additional file 2: Table S2.

### Mice treadmill experiment

Sixteen CL57BL/6 mice about 12 weeks old (±1 week) were purchased from the Experimental Animal Center of Huiji District, Zhengzhou City (Zhengzhou, Henan, China). All mice were housed in the Animal Genetic Engineering Laboratory at Shihezi University. C57BL/6J mice were housed in SPF individually ventilated cages (2 mice per cage) under the controlled room temperature (23°C ± 3°C) and relative humidity (60 ± 10%) conditions, with a reverse light to dark cycle (12:12). C57BL/6J mice were left to acclimate for 1 week and were randomly divided into two groups based on body weight. One group was treated with normal standard diets (Experimental Mice Maintenance Feed (AIN-93), XIETONG SHENGWU, Nanjing, China) containing 3.5% and 0.5% acetate and butyrate (Solarbio, Beijing, China), and the other group was supplemented with an equal amount of sodium chloride (Solarbio, Beijing, China) in the feed as a control. The processed diets were stored in a −20°C freezer, and the diets of all mice were changed daily at 10:00 am. Both groups of mice were given free access to water and diets. For 3 days before starting treatment, mice were acclimated daily to the treadmill (No. XR-PT-10B; Shanghai XinRuan Information Technology Co., Ltd. Shanghai, China) by walking at 10 m/min for 10 min. Exercise capacity was measured after 4 weeks of treatment as previously described [58, 59]. The inclination of the runway was 11°. The exercise regimen was started with shock grid ON at 10 m/min for 30 S; speed was increased by 1 m/3 min up to 20 m/min and then held at 20 m/min until exhaustion. The electric shock intensity was 2mA, and the electric shock tolerance time was 10 s. The exhaustion time of each mouse was recorded for analysis.

### Statistical analysis and graphing

Phylogenetic trees were drawn with GraPhlAn ver. 1.1.3 [60] and the ggtree packages [61] in R ver. 3.6.2 or iTOL. All other statistical analyses were carried out in R ver. 3.6.2. The ComplexHeatmap package [62] was used to visualize all of the heatmaps. Box plots and scatter plots were drawn with the ggplot2 package [63]. Rarefaction analysis was performed to characterize gene richness. Our samples were randomly sampled 100 times with replacement, and the total number of genes that could be identified from these samples was estimated with R ver. 2.15.3 (vegan package) [64].

## Results and discussion

### Samples and metagenomic sequencing data

**Fig. 1:**
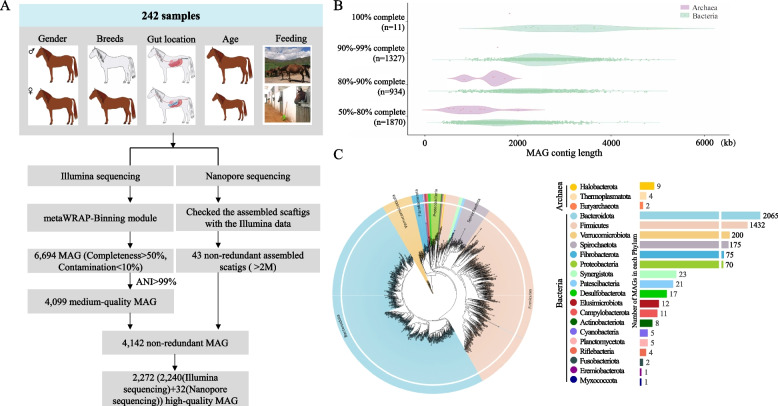
Pipeline for MAG construction and basic information of assembled MAG. **A** Summary of study population characteristics and schematic diagram of the pipeline for constructing MAG from 242 horse metagenome datasets using Illumina and Nanopore sequence data. **B** Distribution of genome completeness and classification of MAG into quality tiers. The abscissa represents the MAG length, and the ordinate represents the MAG completeness. **C** Phylogenetic tree of 4142 MAG from the horse gut, generated by PhyloPhlAn. The coloured circles represent MAG. The legend is arranged in decreasing order (top to bottom) of the number of bacteria detected in the corresponding phyla

To provide a resource for studying the horse gut microbiome, metagenome sequencing was performed on 110 caecal and 132 rectal gut (faeces) samples from a total of 242 horses of different ages (range: 1–11 years), sexes (female, male), and breeds (Thoroughbred, Yili horse, Yili racehorse, Tibetan horse, or Shetland pony) and that were maintained under different diets (Additional file 1: Table S1, Fig. 1A). Using high-throughput sequencing, we obtained 2.267 Tb of Illumina sequencing data from all 242 samples. After quality control, a total of 2.264 Tb of clean, high-quality data remained, with an effective data quality control rate of 99.86% (Additional file 3: Table S3). To evaluate the total number of genes that could be identified from these samples, rarefaction analysis was performed with random sampling 100 times. The rarefaction curve was close to saturation (Additional file 4: Fig. S1), indicating that the sequence data were sufficient for a genomic analysis of the horse gut microbiota and that few novel genes had gone undetected. In addition, for improving the quality of data assembly, we sequenced two samples (HGM35 and HGM77) by using Nanopore sequencing and obtained 0.057 Tb of long-read sequencing data.

### Assembly of 4142 microbial genomes from the horse gut

We used the metaWRAP-Binning module to generate 6408 bins (MaxBin2), 7495 bins (metaBAT2), and 12,415 bins (CONCOCT) (Fig. 1A, left side). After dereplication (average nucleotide identity (ANI) ≤99%) and quality assessment, we obtained a final set of 4099 MAG that met or exceeded the previously established medium-quality criteria (completeness ≥ 50% and contamination ≤ 10%; [65]). An additional 43 genomes were generated from the two sequenced samples (HGM35 and HGM77) via the Nanopore sequencing approach (Fig. 1A, right side). Thus, we assembled a total of 4142 MAG by using Illumina and Nanopore sequencing technologies (Additional file 5: Table S4). Among the 4142 MAG, 2272 were high-quality genomes (defined as > 80% completeness and < 10% contamination [66]) (Fig. 1B); 646 showed > 95% completeness and < 5% contamination; and 46 presented > 97% completeness and 0% contamination (Additional file 6: Fig. S2).

To classify the 4142 MAG, these MAG sequences were aligned with the Genome Taxonomy Database (GTDB). Further analysis showed that 126 of the 4142 MAG were identified at the species level, 3462 were identified at the genus level, 4127 were identified at the family level, 4139 were identified at the order level, and 4142 were identified at the class level. Clustering of MAG with ANI of 99% and 95% for strain-level and species-level genome bins threshold, respectively, we found that 3253 MAG could be considered species-level genomes. All 4142 MAG were classified into GTDB-predicted taxa (Fig. 1C), including 18 bacterial phyla (*n* = 4127 MAG) and 3 archaeal phyla (*n* = 15 MAG). As shown in Fig. 1C, the top 10 bacterial phyla were *Bacteroidota* (2065 MAG), *Firmicutes* (1432 MAG), *Verrucomicrobiota* (200 MAG), *Spirochaetota* (175 MAG), *Fibrobacterota* (75 MAG), *Proteobacteria* (70 MAG), *Synergistota* (23 MAG), *Patescibacteria* (64 MAG), *Desulfobacterota* (17 MAG), *Elusimicrobiota* (12 MAG), and *Campylobacterota* (11 MAG). 4015 of the 4142 MAG (> 96.93%) did not match the reference genomes in the GTDB and therefore represented unknown species or strains identified for the first time in this study (Additional file 7: Table S5). By dereplication with the horse MAG from previous studies [6, 20, 21] based on the 95% ANI level, we found that 3936 MAG were unique in this study (Additional file 8: Table S6). Upon comparing the MAG identified in 242 horses, we found that 36 core MAG were present in at least 90% of samples, including *Bacteroidota* (MAG = 16), *Firmicutes* (MAG = 18) and *Verrucomicrobiota* (MAG = 2) (Additional file 9: Table S7), and these phyla were also present in the reported horse gut metagenomic data [6, 67]. Meanwhile, MAG with a relative abundance of <1% in 90% of the samples were considered rare [68], and a total of 43 MAG were identified as rare (Additional file 10: Table S8).

Among the 15 archaeal MAG obtained in this study, 13 belonged to unknown species. All archaeal MAG were assigned to three phyla: *Halobacterota* (*n* = 9), *Thermoplasmatota* (*n* = 4), and *Euryarchaeota* (*n* = 2). To reveal the potential functions of the archaeal MAG, 6 high-quality archaeal MAG were chosen based on >80% completeness threshold and were further analysed for the presence of methanogenic pathway genes involved in methane production. All 6 high-quality archaeal MAG contained >500 methanogenic genes (Additional file 11: Table S9). Interestingly, MAG23.bin.19 (100% completeness and 0% contamination) contained the most methanogenic genes identified among these archaeal MAG and could utilize all three known pathways (hydrogenotrophic, acetoclastic, and methylotrophic pathways) to produce methane (Additional file 11: Table S9). MAG23.bin.19 was assigned to *Methanobrevibacter smithii*. Previous study reported that *Methanobrevibacter smithii* was the dominant archaeon (representing up to 10% of all anaerobes) in the gut of humans and promoted the production of methane in the human large intestine [69]. Meanwhile, *Methanobrevibacter smithii* is also an important methanogen in the rumen [70]. Together, our results suggest that MAG23.bin.19 may be a major contributor to methane production in horses. The complete genome information of *Methanobrevibacter smithii* may provide novel targets for mitigating methane production, although further experimental analysis is needed to confirm this in the future.

### Assembling the first complete, circularized genomes of 13 unknown species from long-read data

**Fig. 2:**
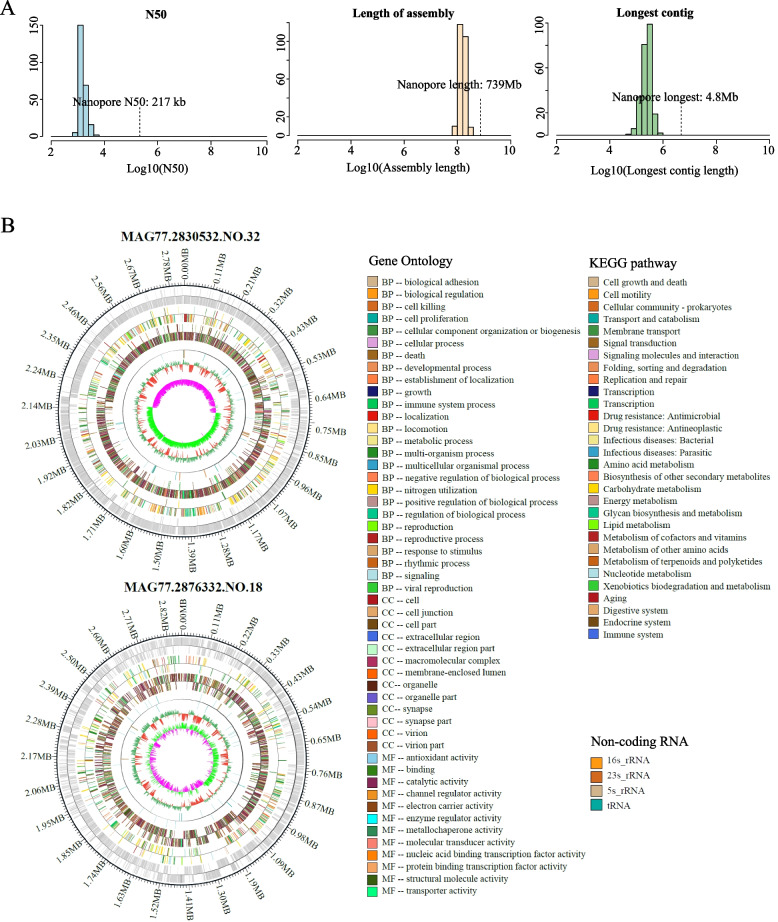
Assembly of the first complete, circularized genomes from long-read data. **A** Statistical distribution of three important indicators (upper, N50 value; middle, total length of components; lower, length of the longest contig) for the Illumina and Nanopore sequence assembly results. Nanopore sequencing indicators are highlighted. **B** Overview of 2 circular genomes. From the outside to the inside, the concentric circles indicate the positional coordinates of the genomic sequence, coding genes, gene annotation, noncoding RNAs, genomic GC content, and genomic GC skew values. Gene annotation: different colours distinguish KEGG and GO annotations. Genome GC content: red indicates that the GC content is less than that of the whole genome; green indicates that the GC content is greater than the average. GC skew value: pink indicates that the G content is less than the C content; light-green indicates that the G content is greater than the C content

To generate complete microbial genomes, two metagenomic samples (HGM35, female; HGM77, male) were subjected to Nanopore sequencing, which produced more than 50.13 Gb of clean data (more than 57.71 Gb of raw data at an efficiency of > 86.85%). The average read length was 55,063 bp, which is superior to the data reported in previous studies [27, 71]. After assembling these long reads, we obtained a sequence that was 739 Mb long, and the N50 value reached 217 kb. As shown in the pipeline diagram (Fig. 1A), we assembled 32 new high-quality MAG that had not previously been identified in public databases. Compared with Illumina data, we observed that Nanopore sequencing data was superior in assembly length and contig length (Fig. 2A, Additional file 5: Table S4).

Long reads can be used to assemble near-complete circular MAG (cMAG) from a single scaffold, which greatly improves the accuracy of the assembly. Among the 32 MAG generated from long reads, 13 were successfully assembled into cMAG, which is an important achievement of long-read assembly (Additional file 12: Table S10). The average completeness and contamination of these cMAG were 93.25% and less than 0.99%, respectively. The cumulative GC skew and genomic GC content were used to verify the assembly quality of the cMAG (Additional file 13: Fig. S3).

These 13 cMAG were further compared with the NR database and the Genome Taxonomy Database Toolkit (GTDB-Tk) to identify homologous species. Among these homologous species, the highest ANI with cMAG was 83.69%. This relatively low ANI indicated the novelty of these cMAG representing the first circularized, complete genomes for respective species. These cMAG contained complete bacterial genome information, including multiple copies of 16S, 5S, and 23S rRNA operons and tRNA operons (Additional file 14: Table S11). We next identified the assembled genomes of the highest quality for further analysis based on contamination and selected MAG77.2830532.NO.32, MAG77.2876332.NO.18, and MAG77.3034135.NO.12, all of which had contamination scores of 0% (Fig. 2B, Additional file 13: Fig. S3, Additional file 12: Table S10). Comparisons with public databases identified MAG77.2830532.NO.32 as *Anaerovibrio lipolyticus* (ANI < 73.90%) and MAG77.2876332.NO.18 as a novel species of *Ruminococcaceae* bacteria (ANI < 71.20%) (Fig. 2B), and MAG77.3034135.NO.12 was identified as an unknown *Ruminococcus* sp. (ANI < 79.17%) [72]. We also searched for full-length 16S sequences in these genomes by comparison against the NCBI database, and the top hits were an uncultured rumen *Anaerovibrio* sp. (97.30% identity), uncultured *Ruminococcus* sp. (95.60% identity), and uncultured *Ruminococcaceae* bacterium (97.30% identity). According to the general standard for 16S rRNA-based taxonomy [73], only MAG77.2876332.NO.18 could be considered a new species, which further suggests the accuracy of the genome sequencing of the microbial taxa. These results suggest that the long-read assembly of MAG not only increased the integrity of the genome assemblies but also revealed previously unresolved genomic features and taxonomic information.

### Hundreds of thousands of novel CAZy

**Fig. 3:**
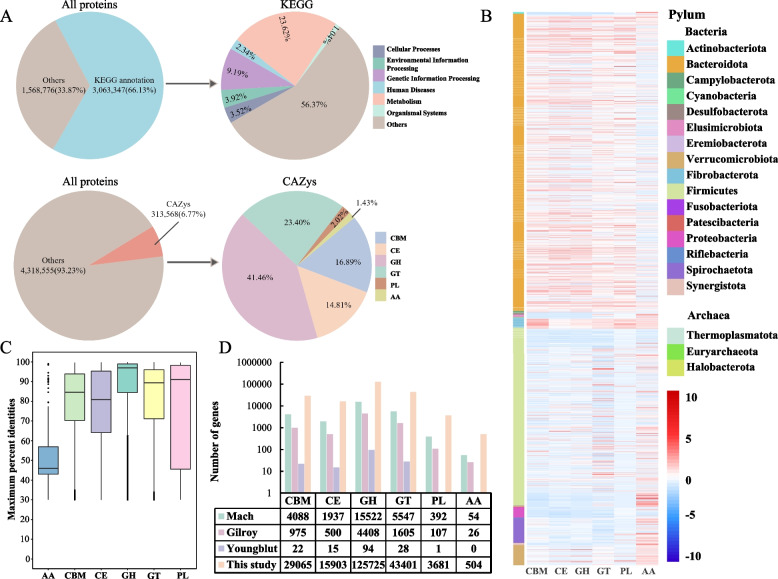
Functional annotation of MAG in the horse gut. **A** Functional annotations of horse microbial proteins. Annotation results obtained using KEGG (upper) and dbCAN2 (lower). **B** Heatmap of the distribution of CAZy. The horizontal axis represents 6 different kinds of CAZy, and the different colours of the vertical axis represent different bacterial taxonomic information. **C** Sequence identity of CAZy in this study with public databases. Center lines indicate the median value; boxes show the interquartile range. The origin at the end of the line represents the extreme value. **D** Comparison of CAZy gene numbers with previous studies. GH, glycoside hydrolase; GT, glycosyltransferase; PL, polysaccharide lyase; CE, carbohydrate esterase; AA, auxiliary activity; CB, carbohydrate binding. The gene number from previous studies and this study were showed in the table below the figure

Next, we analysed the proteomic contents and functions of the horse metagenomes by searching against the KEGG and CAZy databases [74, 75]. The 2272 high-quality MAG contained a total of 4,632,123 predicted proteins. By comparing gene catalogue of this study with that of published horse data reported by Mach et al., Youngblut et al., and Gilroy et al. [6, 20, 67], we found that our data greatly expanded the catalogue of equine gut microbial gene catalogue (Additional file 15: Fig. S4). Among the 4,632,123 predicted proteins, 6.77% (313,568) of which were predicted to have at least one CAZy function (Additional file 16: Table S12). The 313,568 CAZy proteins included 130,001 glycosyl hydrolases (GH), 73,365 glycosyl transferases (GT), 52,961 carbohydrate-binding modules (CBM), 46,437 carboxyesterases (CE), 6320 polysaccharide lyases (PL), and 4484 proteins with auxiliary activity (AA) (Fig. 3A; Additional file 17: Table S13). These proteins were unevenly distributed in the genomes of the taxa that we identified. For example, GH and GT were particularly enriched in Verrucomicrobiota and Firmicutes (Fig. 3B). We further analyse the similarity of the predicted CAZymes against the current CAZy database [27]. Among the 313,568 CAZy proteins, only 126,139 (40.23%) showed highly similar matches with ≥ 95% consistency, indicating that 187,429 of our predicted proteins were novel CAZy (Fig. 3C, Additional file 17: Table S13). Among all the classes of the predicted CAZymes, GH presented the greatest amino acid-level sequence identity (87.72%) with the CAZy in public databases, while AA presented the lowest identity of only 49.18% (Fig. 3C), indicating that a large portion of the diversity of CAZymes is missing from public databases.

We compared the CAZyme profiles of this study with those of prior horse study which was recently made available by Mach et al. [6], Youngblut et al. [20], and Gilroy et al. [67]. We found that our data significantly expand the number of CAZy in the horse gut (Fig. 3D). For these six classes of CAZy, including GT, GH, AA, PL, CE, and CBM, our data expanded the number of CAZy by at least an order of magnitude in the horse gut, although inconsistencies between our study and the prior study related to sample collection or sequencing method that may impact comparisons between these datasets. We further analyse the effect of horse gender or breeds on CAZyme profiles. CAZy gene abundance in the horse gut was significantly associated with sex (*R*^2^ = 0.04, *P* = 0.032) and breed (*R*^2^ = 0.2107, *P* = 0.001).

In addition, we detected 4492 polysaccharide utilization loci (PUL) among 556 *Bacteroides* species (Additional file 18: Table S14), which can degrade a variety of carbohydrate substrates in animal digestive systems [76]. Most of the 556 *Bacteroides* genomes had at least one PUL; *Bacteroides fragilis* (MAG117.bin.13), in particular, contained up to 48 PUL. In total, the identification of these novel CAZy and potentially cellulose-degrading bacteria will facilitate a better understanding of carbohydrate metabolism in horse gut and provide a rich source of novel enzymes and microbes for fermentation biotechnology industries [77, 78].

### The horse gut as a reservoir of antibiotic resistance genes

**Fig. 4:**
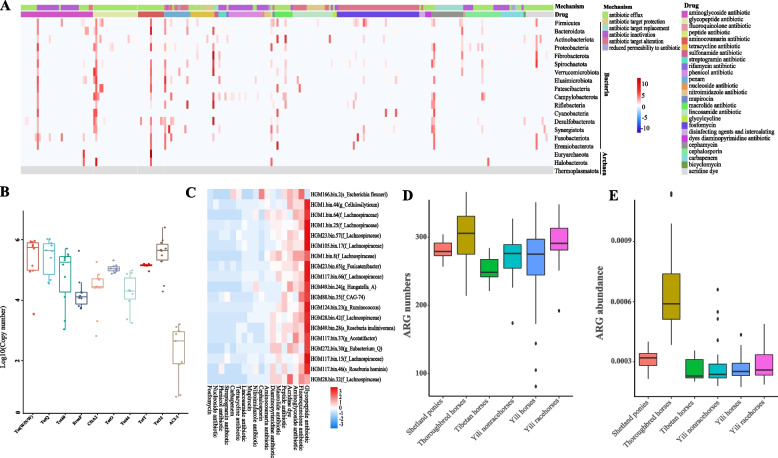
The horse gut as a reservoir of antibiotic resistance genes. **A** Heatmap of the ARG distribution. Colour from blue to red represents an increasing number of ARG. The colour band at the top of the heatmap represents the resistance mechanism corresponding to each column of ARG. The second colour band represents the resistance drug corresponding to each column of ARG. **B** Real-time RT–PCR analysis results of 10 randomly selected ARG. The ordinate represents the logarithmic change in ARG copy number. **C** Heatmap of resistance-associated drug classes in the top 20 MAG with the number of ARG. Colours from blue to red represent an increasing number of ARG. On the right side of the heat map is the ID and taxonomic information of MAG. These MAG are arranged from top to bottom according to the number of encoded ARG. **D**, **E** Gene counts (**D**) or relative abundance (**E**) of ARG in horses gut microbes of different breeds. The abscissa represents the 5 horse breeds

To characterize horse intestinal antibiotic resistance genes (ARG), we examined the distribution of ARG in 2272 high-quality MAG among 242 horse gut samples. We identified a total of 266 unique types of ARG across 25 drug resistance classes in the horse gut MAG (Fig. 4A). The number of both ARG and drug resistance classes are fewer than those reported in pig and bovine gut [79, 80], which may be related to genetics, diet, and exposure to antibiotics in life. *Firmicutes* and *Bacteroidota* were the bacterial groups with the greatest numbers of ARG, and we also predicted ARG in three archaeal taxa, including *Euryarchaeota* and *Halobacterota* (Fig. 4A, Additional file 19: Table S15). Aminoglycoside, aminocoumarin, and tetracycline ARG were prevalent in the intestines of horses (Fig. 4A, Additional file 19: Table S15), which was consistent with previous ARG studies in the gut microbes of cattle, sheep, pig, chicken, and horses [20, 81, 82]. However, the aminoglycoside resistance genes were the most abundant ARG in the intestines of horses. The high abundance of aminoglycoside resistance genes has a potential negative impact on horse health given that aminoglycoside was widely used to treat respiratory diseases, septic peritonitis, acute febrile diarrhoea, and cellulitis in horses [83, 84].

Furthermore, a total of 2194 MAG (96.57%) contained five or more ARG (Additional file 20: Table S16), suggesting that ARG are widespread in the horse gut microbiome. We verified the transcriptional activity of these ARG by real-time RT-PCR using 10 randomly chosen ARG, and the results indicated that these predicted genes have real drug resistance functions (Fig. 4B, Additional file 21: Fig. S5). Surprisingly, an *Escherichia coli* strain (MAG166.bin.2) was found to contain 82 unique types of ARG belonging to 13 drug resistance classes (Fig. 4C, Additional file 20: Table S16). The strain contained a variety of resistance genes against fluoroquinolone antibiotics, cephalosporins, acridine dyes, carbapenems, aminoglycoside antibiotics, peptide antibiotics, and tetracycline antibiotics commonly used in livestock production. Considering the common pathogenicity of *E. coli*, this finding suggests that this strain may be a potential drug-resistant superbug. In addition, we found a large number of ARG in MAG49.bin.28 (*Roseburia),* MAG250.bin.22 (*Prevotella*), and MAG77.2754209.NO.29 (*Akkermansia*), members of which are widely reported to have probiotic effects [85–87] (Additional file 20: Table S16). Although antibiotic resistance could be used for facilitating the future isolation and culture of these strains, we may also consider the potential harm caused by these ARG to the host gut microbiome.

Then, the pair-wise comparisons of ARG prevalence in the gut of six populations (Yili horses, Thoroughbred, Shetland ponies, Tibetan horses, Yili nonracehorses, and Yili racehorses) were performed. Although the Yili horses had the largest sample size, the number and abundance of ARG in the Yili horses were not the largest. However, Thoroughbred horses had the highest abundance and number of ARG, which was more than two-fold times than that of Tibetan horses (Fig. 4D, E, Additional file 22: Table S17). Tibetan horses have the lowest abundance and number of ARG in the gut, and the horses graze freely on the Qinghai-Tibet Plateau with rare exposure to antibiotics. In addition, although genetic backgrounds, training patterns, and breeding sites of Yili racehorses, Yili horses, and Shetland ponies were different, the abundance and quantity of ARG in the guts of these horses were similar. Among these six breeds, Thoroughbred horses are subjected to commercially formulated feeds, antibiotic administration, stabling, and confinement conditions throughout the life cycle, which may be one of the reasons for the high antibiotic abundance of ARG in Thoroughbred populations [79].

### Novel insights into potential performance-enhancing microbes in racehorses

**Fig. 5:**
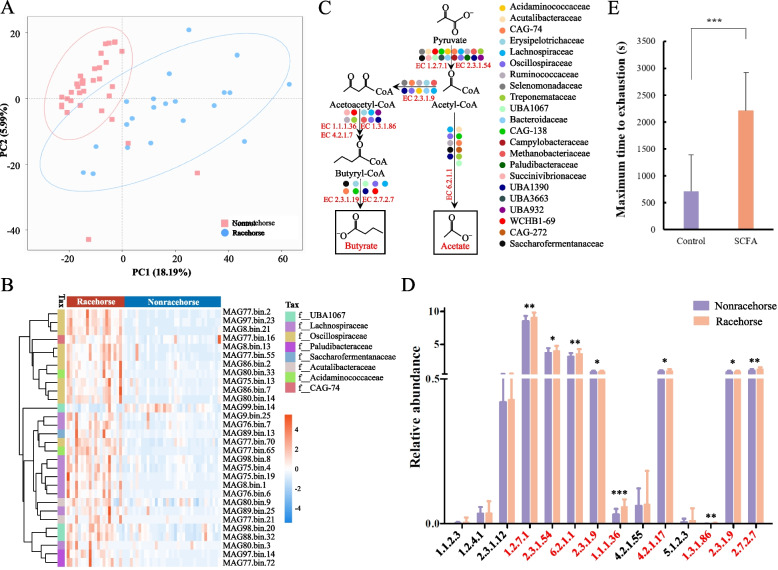
Novel insights into potential performance-enhancing microbes in racehorses. **A** Differences in the gut microbiota of racehorses and nonracehorses were visualized by PCoA. **B** Top 30 differentially abundant MAG in racehorses and nonracehorses. These MAG belong to different tax of bacteria presented on the left column. Colours from blue to red represent an increasing abundance of MAG. **C** Bacteria enriched in the racehorse contained key enzyme in each step of the acetate and butyrate synthesis pathway. Different colours represent different types of bacteria that contain the enzymes necessary for the pathway. The metabolic pathway and EC numbers obtained from KEGG database. **D** The bar chart shows the differences in key enzymes involved in acetate and butyrate synthesis between racehorses and nonracehorses. Enzymes (EC number in red) that produced acetate and butyrate are significantly enriched in the racehorse. EC numbers on horizontal axis were obtained from KEGG database. **E** Acetate and butyrate treatment significantly improve the exercise performance of mice. The plot shows each mouse as an individual point, and the central bar representing the mean time (*n* = 8). *P < 0.05, **P < 0.01, ***P < 0.001

To reveal the link between the gut microbiota and exercise performance in racehorses, we examined the differences between the microbiomes of elite racehorses (*n* = 21, Yili racehorses) and a group of age-matched nonracehorses horses (*n* = 35, Yili horses). Principal coordinate analysis (PCoA) revealed that the microbial compositions of the racehorses and nonracehorses were distinctly separated (Fig. 5A). Further analysis of the differences in the composition of the gut microbiomes of the two groups at the species level was performed. The MAG from *Prevotella* (MAG36.bin.6, MAG266.bin.12, MAG146.bin.68, MAG122.bin.6, MAG120.bin.60) [88], *Lachnospiraceae* (MAG252.bin.16, MAG32.bin.44) [89], *Phascolarctobacterium* (MAG20.bin.3, MAG19.bin.10, MAG23.bin.66, MAG3.bin.34, MAG58.bin.49, MAG23.bin.2, MAG35.bin.4, MAG80.bin.33, MAG240.bin.26, MAG77.bin.65, MAG104.bin.14, MAG148.bin.27) [90], *Oscillospiraceae* (MAG59.bin.38) [91], *Eubacterium* (MAG77.bin.21) [92], and *Ruminococcus* (MAG73.bin.20, MAG28.bin.65) [93] showed higher abundances in the guts of elite racehorses than in those of nonracehorses (Fig. 5B, Additional file 23: Table S18). *Lachnospiraceae* and *Ruminococcus* were the highly abundant microbes in racehorse, which is in agreement with a study in endurance horses by Plancade et al. [14]. Interestingly, *Lachnospiraceae*, *Oscillospiraceae*, and *Ruminococcus*, defined as performance-associated bacteria, have been found to be enriched in the microbiomes of human athletes, suggesting that the performance-associated bacteria may be, at least in part, conserved across animals and human [94].

To further explore the functions of the differentially abundant bacteria, we compared the metabolic pathways of the microbiome between the racehorses group and nonracehorses group. As shown in Fig. 5C, *Lachnospiraceae*, *Phascolarctobacterium*, *Oscillospiraceae*, *Eubacterium*, *Ruminococcus*, *Campylobacteraceae*, *Methanobacteriaceae*, *Succinivibrionaceae*, *Bacteroidaceae*, *Erysipelotrichaceae*, and *Treponemataceae*, which were enriched in the racehorses (Fig. 5B, Additional file 23: Table S18), contained many key enzymes in the acetate and butyrate synthesis pathways. The key enzymes (EC 1.2.7.1, EC 2.3.1.54, EC 6.2.1.1, EC2.3.1.9, EC 1.1.1.36, EC 4.2.1.17, EC 1.3.1.86, EC 2.3.1.9, and EC 2.7.2.7) in the acetate and butyrate synthesis pathways were significantly enriched in the racehorses gut when compared with nonracehorses (Fig. 5D). More importantly, 36 MAG (e.g. MAG15.bin.4 belonging to *Lachnospiraceae*, MAG122.bin.15 belonging to *Oscillospirace*ae, MAG28.bin.65 belonging to *Ruminococcus*, and MAG17.bin.19 belonging to *Treponema*) were higher in racehorses than that in nonracehorses (Additional file 24: Fig. S6). The enrichment of *Treponema* in racehorses is consistent with previous studies performed in endurance horses by Plancade et al. [14] and in Standardbred racehorses by Janabi et al. [19] that showed that training led to an increase in *Treponema*. These MAG enriched in Yili racehorses contained every gene in a major pathway for producing acetate and butyrate (Additional file 24: Fig. S6). These results suggest these microbes enriched in the racehorses may synthesize more acetate and butyrate in the gut. However, the differences observed here were likely due to the different diets, environment, or exercise of racehorses and nonracehorses. Future studies is needed to confirm this.

Acetate and butyrate have been shown to modulate skeletal muscle function and exercise capacity [93, 95–97]. Several groups have reported that continuous supplementation of acetate alone or together with butyrate improves endurance performance and muscular strength [98, 99]. We also confirmed that infusion of the SCFA (a mixture of acetate and butyrate) into mice resulted in significant increase of maximum time to exhaustion (Fig. 5E). Mice treated with oral SCFA infusion, on average, showed approximately 87.23% increase in run times when compared with the saline vehicle group (*n* = 8). Although it has been reported that oral acetic acid can serve as an important energy source for skeletal muscle at rest in horses [95, 96], whether or not SCFA can enhance athletic performance in horses needs to be in-depth studied in the future.

## Conclusions

We present the large-scale metagenomic sequencing dataset and reference genome assembly for the horse gut microbiome, which will improve the ability to perform taxonomic grouping and metagenomic and metatranscriptomic analyses for future microbiome studies of horses. High abundance and diversity of ARG were identified in the MAG, which showed the horse gut as a reservoir of antibiotic resistance genes. In addition, by using the assembled genome to mine the functions of the horse gut microbiome, 36 MAG (e.g. belonging to *Lachnospiraceae*, *Oscillospiraceae*, and *Ruminococcus*) was observed to be enriched in the racehorses. These bacteria enriched in racehorses may produce more acetate and butyrate, presenting potential for greater amount of short-chain fatty acids available to fuel athletic performance. These acetate and butyrate-producing microbes are expected to be used as biomarkers for identifying or selecting endurance racehorses and may be developed into probiotics that are used for promoting horse exercise and health in the future. Our study provides exhaustive reference genomic datasets for the horse gut microbiota, and our results emphasize the complex interactions between the host and the gut microbiota.

## Supplementary Information

**Additional file 1: Table S1.** All detailed information on experimental horses and samples.

**Additional file 2: Table S2.** Details of the primers of the 10 ARG used for real-time PCR.

**Additional file 3: Table S3.** Statistics and quality control information for the sequence data.

**Additional file 4: Figure S1.** Rarefaction curve of the gene numbers in our samples. The curve nearly plateaus when sufficient sequence data are included, with few novel genes being left undetected.

**Additional file 5: Table S4.** Information on 4142 MAG obtained from the gut of horse using Illumina and Nanopore sequencing technologies.

**Additional file 6: Figure S2.** Assessment of the degree of contamination and integrity of 2272 high-quality MAG in the horse gut. The grey circles indicate 80–90% genome integrity with 5–10% contamination; the red circles indicate > 90% genome integrity with < 5% contamination.

**Additional file 7: Table S5.** Comparison of 4142 MAG with the public database.

**Additional file 8: Table S6.** Check the uniqueness of the MAG in this study by deduplicating the MAG reported in previous studies.

**Additional file 9: Table S7.** Detailed description of the 36 core MAG. Core MAG were present in at least 90% samples.

**Additional file 10: Table S8.** Abundance of 4142 MAG in 242 samples.

**Additional file 11: Table S9.** All 6 archaeal MAG contained hundreds of methanogenic genes.

**Additional file 12: Table S10.** Details of the MAG assembled by Nanopore sequencing that are expected to be circular contigs.

**Additional file 13: Figure S3.** Overview of 11 circular genomes. From the outside to the inside, the concentric circles indicate the positional coordinates of the genomic sequence, coding genes, gene annotation, noncoding RNAs, genomic GC content, and genomic GC skew values. Gene annotation: different colours are used to distinguish different terms of the KEGG and GO annotations. Genome GC content: the inner, red part indicates that the GC content of the region is less than the average GC content of the whole genome; the outer, green part indicates that the GC content of the region is greater than the average value. GC skew value: the inner, pink part indicates that the G content in the area is less than the C content; the outer, light-green part indicates that the G content in the area is greater than the C content.

**Additional file 14: Table S11.** 16S rRNA gene data in the complete genomes assembled by Nanopore sequencing.

**Additional file 15: Figure S4.** Venn plots of the gene catalogues of this study and previous published horse study. Data for published articles are marked with author names.

**Additional file 16: Table S12.** Analysis of proteins functions for the 2272 MAG.

**Additional file 17: Table S13.** Detailed information on the six active polysaccharide-degrading enzymes (GH, GT, PL, CE, CBM, and AA) among the proteins encoded by the 2272 genomes.

**Additional file 18: Table S14.** Details of 4492 PUL contained in 556 *Bacteroides* MAG.

**Additional file 19: Table S15.** ARG encoded by horse intestinal MAG were identified into 25 resistance classes.

**Additional file 20: Table S16.** 266 ARG predicted in 2272 MAG.

**Additional file 21: Figure S5.** RT–PCR amplification of 10 randomly selected ARG. M is the size marker (Takara DL500: 500 bp, 400 bp, 300 bp, 200 bp, 150 bp, 100 bp, and 50 bp), and N is the negative control.

**Additional file 22: Table S17.** Abundance of antibiotic resistance genes in 242 samples.

**Additional file 23: Table S18.** The differences of microbial composition in racehorses and nonracehorses.

**Additional file 24: Figure S6.** The racehorse gut is rich in microbes that can synthesize acetate and butyrate. The microbes enriched in racehorses contain a large number of enzymes that synthesize acetate and butyrate. At the bottom of the heatmap are the difference MAG ID, and the top are the taxonomic information of the MAG.

## Data Availability

All of the original sequences obtained in this work have been deposited in the National Center for Biotechnology Information (NCBI) under project number PRJEB871798. The MAG sequences, the gene catalogue, and source code are available at Zenodo (10.5281/zenodo.7240014). All of the data can also be obtained from the corresponding author upon reasonable request or from the included supplementary information files.

## References

[1] Wade CM, Giulotto E, Sigurdsson S, Zoli M, Gnerre S, Imsland F (2009). Genome sequence, comparative analysis, and population genetics of the domestic horse. Science.

[2] Jastrzębska E, Daszkiewicz T, Gorecka-Bruzda AL, Feliś DI (2019). Current situation and prospects for the horse meat market in Poland and the world. Med Weter.

[3] Cross P (2019). Global Horse statistics internal 02 2019.

[4] Huntley NF, Naumann HD, Kenny AL, Kerley MS (2017). Black rhinoceros (Diceros bicornis) and domestic horse (*Equus caballus*) hindgut microflora demonstrate similar fermentation responses to grape seed extract supplementation in vitro. J Anim Physiol Anim Nutr.

[5] Yang C (2018). Acetogen communities in the gut of herbivores and their potential role in syngas fermentation. Fermentation.

[6] Mach N, Midoux C, Leclercq S, Pennarun S, Le Moyec L, Rué O, et al. The first horse gut microbiome gene catalog reveals that rare microbiome ensures better cardiovascular fitness in endurance horses. bioRxiv. 2022. 10.1101/2022.01.24.477461.

[7] Edwards JE, Shetty SA, Van Den Berg P, Burden F, Van Doorn DA, Pellikaan WF (2020). Multi-kingdom characterization of the core equine fecal microbiota based on multiple equine (sub) species. Anim Microbiome.

[8] Dougal K, Harris PA, Edwards A, Pachebat JA, Blackmore TM, Worgan HJ (2012). A comparison of the microbiome and the metabolome of different regions of the equine hindgut. FEMS Microbiol Ecol.

[9] Kauter A, Epping L, Semmler T, Antao EM, Kannapin D, Stoeckle SD (2019). The gut microbiome of horses: current research on equine enteral microbiota and future perspectives. Anim Microbiome.

[10] Costa MC, Arroyo LG, Allen-Vercoe E, Stämpfli HR, Kim PT, Sturgeon A, et al. Comparison of the fecal microbiota of healthy horses and horses with colitis by high throughput sequencing of the V3-V5 region of the 16S rRNA gene. PLoS One. 2012. 10.1371/journal.pone.0041484. 10.1371/journal.pone.0041484 PMC3409227 22859989

[11] Feary DJ, Abraham S, Woolford L, Trott DJ (2013). Identification of A ctinomyces denticolens as a cause of a soft tissue abscess in a horse. Aust Vet J.

[12] Schoster A, Staempfli HR, Guardabassi LG, Jalali M, Weese JS (2017). Comparison of the fecal bacterial microbiota of healthy and diarrheic foals at two and four weeks of life. BMC Vet Res.

[13] Mach N, Foury A, Kittelmann S, Reigner F, Moroldo M, Ballester M (2017). The effects of weaning methods on gut microbiota composition and horse physiology. Front Physiol.

[14] Plancade S, Clark A, Philippe C, Helbling JC, Moisan MP, Esquerré D (2019). Unraveling the effects of the gut microbiota composition and function on horse endurance physiology. Sci Rep.

[15] Mach N, Moroldo M, Rau A, Lecardonnel J, Le Moyec L, Robert C (2021). Understanding the holobiont: crosstalk between gut microbiota and mitochondria during long exercise in horse. Front Mol Biosci.

[16] Salem SE, Maddox TW, Antczak P, Ketley JM, Williams NJ, Archer DC (2019). Acute changes in the colonic microbiota are associated with large intestinal forms of surgical colic. BMC Vet Res.

[17] Weese JS, Holcombe SJ, Embertson RM, Kurtz KA, Roessner HA, Jalali M (2015). Changes in the faecal microbiota of mares precede the development of post partum colic. Equine Vet J.

[18] Górniak W, Cholewińska P, Szeligowska N, Wołoszyńska M, Soroko M, Czyż K (2021). Effect of intense exercise on the level of bacteroidetes and Firmicutes phyla in the digestive system of thoroughbred racehorses. Animals.

[19] Janabi AHD, Biddle AS, Klein D, McKeever KH (2016). Exercise training-induced changes in the gut microbiota of Standardbred racehorses. Comp Exerc Physiol.

[20] Ang L, Vinderola G, Endo A, Kantanen J, Jingfeng C, Binetti A (2022). Gut microbiome characteristics in feral and domesticated horses from different geographic locations. Commun Biol.

[21] Youngblut ND, Reischer GH, Dauser S, Maisch S, Walzer C, Stalder G (2021). Vertebrate host phylogeny influences gut archaeal diversity. Nat Microbiol.

[22] Lewis RW, Islam AA, Dilla-Ermita CJ, Hulbert SH, Sullivan TS (2019). High-throughput Siderophore screening from environmental samples: plant tissues, bulk soils, and rhizosphere soils. J Vis Exp.

[23] Pasolli E, Asnicar F, Manara S, Zolfo M, Karcher N, Armanini F (2019). Extensive unexplored human microbiome diversity revealed by over 150,000 genomes from metagenomes spanning age, geography, and lifestyle. Cell.

[24] Stewart RD, Auffret MD, Warr A, Wiser AH, Press MO, Langford KW (2018). Assembly of 913 microbial genomes from metagenomic sequencing of the cow rumen. Nat Commun.

[25] Glendinning L, Stewart RD, Pallen MJ, Watson KA, Watson M (2020). Assembly of hundreds of novel bacterial genomes from the chicken caecum. Genome Biol.

[26] Wang W, Hu H, Zijlstra RT, Zheng J, Gänzle MG (2019). Metagenomic reconstructions of gut microbial metabolism in weanling pigs. Microbiome.

[27] Stewart RD, Auffret MD, Warr A, Walker AW, Roehe R, Watson M (2019). Compendium of 4,941 rumen metagenome-assembled genomes for rumen microbiome biology and enzyme discovery. Nat Biotechnol.

[28] Griffith GW, Ozkose E, Theodorou MK, Davies DR (2009). Diversity of anaerobic fungal populations in cattle revealed by selective enrichment culture using different carbon sources. Fungal Ecol.

[29] Karlsson FH, Tremaroli V, Nookaew I, Bergström G, Behre CJ, Fagerberg B (2013). Gut metagenome in European women with normal, impaired and diabetic glucose control. Nature.

[30] Qin N, Yang F, Li A, Prifti E, Chen Y, Shao L (2014). Alterations of the human gut microbiome in liver cirrhosis. Nature.

[31] De Coster W, D’Hert S, Schultz DT, Cruts M, Van Broeckhoven C (2018). NanoPack: visualizing and processing long-read sequencing data. Bioinformatics.

[32] Chaisson MJ, Tesler G (2012). Mapping single molecule sequencing reads using Basic Local Alignment with Successive Refinement (BLASR): theory and application. BMC Bioinformatics.

[33] Luo R, Liu B, Xie Y, Li Z, Huang W, Yuan J (2012). SOAPdenovo2: an empirically improved memory-efficient short-read de novo assembler. GigaScience.

[34] Uritskiy GV, DiRuggiero J, Taylor J (2018). MetaWRAP-a flexible pipeline for genome-resolved metagenomic data analysis. Microbiome.

[35] Sun X, Ward BB (2021). Novel metagenome-assembled genomes involved in the nitrogen cycle from a Pacific oxygen minimum zone. ISME Commun.

[36] Parks DH, Imelfort M, Skennerton CT, Hugenholtz P, Tyson GW (2015). CheckM: assessing the quality of microbial genomes recovered from isolates, single cells, and metagenomes. Genome Res.

[37] Kolmogorov M, Yuan J, Lin Y, Pevzner PA (2019). Assembly of long, error-prone reads using repeat graphs. Nat Biotechnol.

[38] Sunagawa S, Coelho LP, Chaffron S, Kultima JR, Labadie K, Salazar G (2015). Structure and function of the global ocean microbiome. Science.

[39] Asnicar F, Thomas AM, Beghini F, Mengoni C, Manara S, Manghi P (2020). Precise phylogenetic analysis of microbial isolates and genomes from metagenomes using PhyloPhlAn 3.0. Nat Commun.

[40] Lagesen K, Hallin P, Rødland EA, Stærfeldt HH, Rognes T, Ussery DW (2007). RNAmmer: consistent and rapid annotation of ribosomal RNA genes. Nucleic Acids Res.

[41] Chan PP, Lowe TM, Kollmar M (2019). tRNAscan-SE: searching for tRNA genes in genomic sequences. Gene prediction. Methods in molecular biology.

[42] Pritchard L, Glover RH, Humphris S, Elphinstone JG, Toth IK (2016). Genomics and taxonomy in diagnostics for food security: soft-rotting enterobacterial plant pathogens. Anal Methods.

[43] Zhu W, Lomsadze A, Borodovsky M (2010). Ab initio gene identification in metagenomic sequences. Nucleic Acids Res.

[44] Li W, Godzik A (2006). Cd-hit: a fast program for clustering and comparing large sets of protein or nucleotide sequences. Bioinformatics.

[45] Li J, Jia H, Cai X, Zhong H, Feng Q, Sunagawa S (2014). An integrated catalog of reference genes in the human gut microbiome. Nat Biotechnol.

[46] Arumugam M, Raes J, Pelletier E, Le Paslier D, Yamada T, Mende DR (2011). Enterotypes of the human gut microbiome. Nature.

[47] Huson DH, Auch AF, Qi J, Schuster SC (2007). MEGAN analysis of metagenomic data. Genome Res.

[48] Li J, Zhao F, Wang Y, Chen J, Tao J, Tian G (2017). Gut microbiota dysbiosis contributes to the development of hypertension. Microbiome.

[49] Zeller G, Tap J, Voigt AY, Sunagawa S, Kultima JR, Costea PI (2014). Potential of fecal microbiota for early-stage detection of colorectal cancer. Mol Syst Biol.

[50] Chaumeil PA, Mussig AJ, Hugenholtz P, Parks DH (2019). Gtdb-tk: a toolkit to classify genomes with the genome taxonomy database. Bioinformatics.

[51] Bairoch A, Apweiler R, Wu CH, Barker WC, Boeckmann B, Ferro S (2005). The universal protein resource (UniProt). Nucleic Acids Res.

[52] Feng Q, Liang S, Jia H, Stadlmayr A, Tang L, Lan Z (2015). Gut microbiome development along the colorectal adenoma–carcinoma sequence. Nat Commun.

[53] Zhang H, Yohe T, Huang L, Entwistle S, Wu P, Yang Z (2018). dbCAN2: a meta server for automated carbohydrate-active enzyme annotation. Nucleic Acids Res.

[54] Cantarel BL, Coutinho PM, Rancurel C, Bernard T, Lombard V, Henrissat B (2018). The Carbohydrate-Active EnZymes database (CAZy): an expert resource for glycogenomics. Nucleic Acids Res.

[55] Dixon P (2003). VEGAN, a package of R functions for community ecology. J Veg Sci.

[56] Martínez JL, Coque TM, Baquero F (2015). What is a resistance gene? Ranking risk in resistomes. Nat Rev Microbiol.

[57] Mcarthur AG, Waglechner N, Nizam F, Yan A, Azad MA, Baylay AJ (2013). The comprehensive antibiotic resistance database. Antimicrob Agents Chemother.

[58] Smith PM, Howitt MR, Panikov N, Michaud M, Gallini CA, Bohlooly-y M (2013). The microbial metabolites, short-chain fatty acids, regulate colonic Treg cell homeostasis. Science.

[59] Fachi JL, de Souza Felipe J, Pral LP, da Silva BK, Corrêa RO, de Andrade MCP (2019). Butyrate protects mice from Clostridium difficile-induced colitis through an HIF-1-dependent mechanism. Cell Rep.

[60] Asnicar F, Weingart G, Tickle TL, Huttenhower C, Segata N (2015). Compact graphical representation of phylogenetic data and metadata with GraPhlAn. PeerJ.

[61] Yu G, Smith DK, Zhu H, Guan Y, Lam TTY (2017). ggtree: an R package for visualization and annotation of phylogenetic trees with their covariates and other associated data. Methods Ecol Evol.

[62] Gu Z, Eils R, Schlesner M (2016). Complex heatmaps reveal patterns and correlations in multidimensional genomic data. Bioinformatics.

[63] Wickham H (2011). ggplot2. Wiley Interdiscip Rev Comput Stat.

[64] Hsieh TC, Ma KH, Chao A (2016). iNEXT: an R package for rarefaction and extrapolation of species diversity (H ill numbers). Methods Ecol Evol.

[65] Pan S, Zhu C, Zhao XM, Coelho LP (2022). A deep siamese neural network improves metagenome-assembled genomes in microbiome datasets across different environments. Nat Commun.

[66] Bowers RM, Kyrpides NC, Stepanauskas R, Harmon-Smith M, Doud D, Reddy TBK (2017). Minimum information about a single amplified genome (MISAG) and a metagenome-assembled genome (MIMAG) of bacteria and archaea. Nat Biotechnol.

[67] Gilroy R, Leng J, Ravi A, Adriaenssens EM, Oren A, Baker D (2022). Metagenomic investigation of the equine faecal microbiome reveals extensive taxonomic diversity. PeerJ.

[68] Peng X, Wilken SE, Lankiewicz TS, Gilmore SP, Brown JL, Henske JK (2021). Genomic and functional analyses of fungal and bacterial consortia that enable lignocellulose breakdown in goat gut microbiomes. Nat Microbiol.

[69] Chibani CM, Mahnert A, Borrel G, Almeida A, Werner A, Brugère JF (2022). A catalogue of 1,167 genomes from the human gut archaeome. Nat Microbiol.

[70] Zhou M, Chung YH, Beauchemin KA, Holtshausen L, Oba M, McAllister TA (2011). Relationship between rumen methanogens and methane production in dairy cows fed diets supplemented with a feed enzyme additive. J Appl Microbiol.

[71] Moss EL, Maghini DG, Bhatt AS (2020). Complete, closed bacterial genomes from microbiomes using nanopore sequencing. Nat Biotechnol.

[72] Parks DH, Chuvochina M, Chaumeil PA, Rinke C, Mussig AJ, Hugenholtz P (2020). A complete domain-to-species taxonomy for Bacteria and Archaea. Nat Biotechnol.

[73] Karst SM, Dueholm MS, McIlroy SJ, Kirkegaard RH, Nielsen PH, Albertsen M (2018). Retrieval of a million high-quality, full-length microbial 16S and 18S rRNA gene sequences without primer bias. Nat Biotechnol.

[74] Kanehisa M, Goto S, Sato Y, Kawashima M, Furumichi M, Tanabe M (2014). Data, information, knowledge and principle: back to metabolism in KEGG. Nucleic Acids Res.

[75] Mistry J, Finn RD, Eddy SR, Bateman A, Punta M (2013). Challenges in homology search: HMMER3 and convergent evolution of coiled-coil regions. Nucleic Acids Res.

[76] Cantarel BL, Coutinho PM, Rancurel C, Bernard T, Lombard V, Henrissat B (2009). The Carbohydrate-Active EnZymes database (CAZy): an expert resource for Glycogenomics. Nucleic Acids Res.

[77] Senoura T, Taguchi H, Ito S, Hamada S, Matsui H, Fukiya S (2009). Identification of the cellobiose 2-epimerase gene in the genome of Bacteroides fragilis NCTC 9343. Biosci Biotechnol Biochem.

[78] Cheng Y, Shi Q, Sun R, Liang D, Li Y, Li Y (2018). The biotechnological potential of anaerobic fungi on fiber degradation and methane production. World J Microbiol Biotechnol.

[79] Zhou Y, Fu H, Yang H, Wu J, Chen Z, Jiang H (2022). Extensive metagenomic analysis of the porcine gut resistome to identify indicators reflecting antimicrobial resistance. Microbiome.

[80] Zhu Z, Cao M, Wang W, Zhang L, Ma T, Liu G (2021). Exploring the prevalence and distribution patterns of antibiotic resistance genes in bovine gut microbiota using a metagenomic approach. Microb Drug Resist.

[81] Sabino YNV, Santana MF, Oyama LB, Santos FG, Moreira AJS, Huws SA (2019). Characterization of antibiotic resistance genes in the species of the rumen microbiota. Nat Commun.

[82] Ma L, Xia Y, Li B, Yang Y, Li LG, Tiedje JM (2016). Metagenomic assembly reveals hosts of antibiotic resistance genes and the shared resistome in pig, chicken, and human feces. Environ Sci Technol.

[83] Redpath A, Hallowell GD, Bowen IM (2021). Use of aminoglycoside antibiotics in equine clinical practice; a questionnaire-based study of current use. Vet Med Sci.

[84] Zhang L, Li H, Gao J, Gao J, Wei D, Qi Y (2020). Identification of drug-resistant phenotypes and resistance genes in Enterococcus faecalis isolates from animal feces originating in Xinjiang, People’s Republic of China. Can J Anim Sci.

[85] Tamanai-Shacoori Z, Smida I, Bousarghin L, Loreal O, Meuric V, Fong SB (2017). Roseburia spp.: a marker of health?. Future Microbiol.

[86] Gálvez EJC, Iljazovic A, Amend L, Lesker TR, Renault T, Thiemann S (2020). Distinct polysaccharide utilization determines interspecies competition between intestinal Prevotella spp. Cell Host Microbe.

[87] Hasani A, Ebrahimzadeh S, Hemmati F, Khabbaz A, Hasani A, Gholizadeh P (2021). The role of Akkermansia muciniphila in obesity, diabetes and atherosclerosis. J Med Microbiol.

[88] Mohr AE, Jäger R, Carpenter KC, Kerksick CM, Purpura M, Townsend JR (2020). The athletic gut microbiota. J Int Soc Sports Nutr.

[89] Garber A, Hastie P, Murray JA (2020). Factors influencing equine gut microbiota: current knowledge. J Equine Vet Sci.

[90] Cella V, Bimonte VM, Sabato C, Paoli A, Baldari C, Campanella M (2021). Nutrition and physical activity-induced changes in gut microbiota: possible implications for human health and athletic performance. Foods.

[91] Jie Z, Liang S, Ding Q, Li F, Sun X, Lin Y (2021). Dairy consumption and physical fitness tests associated with fecal microbiome in a Chinese cohort. Med Microecol.

[92] Scheiman J, Luber JM, Chavkin TA, MacDonald T, Tung A, Pham LD (2019). Meta-omics analysis of elite athletes identifies a performance-enhancing microbe that functions via lactate metabolism. Nat Med.

[93] Mach N, Lansade L, Bars-Cortina D, Dhorne-Pollet S, Foury A, Moisan MP (2021). Gut microbiota resilience in horse athletes following holidays out to pasture. Sci Rep.

[94] Han M, Yang K, Yang P, Zhong C, Chen C, Wang S (2020). Stratification of athletes’ gut microbiota: the multifaceted hubs associated with dietary factors, physical characteristics and performance. Gut Microbes.

[95] Waller AP, Geor RJ, Spriet LL, Heigenhauser GJ, Lindinger MI (2009). Oral acetate supplementation after prolonged moderate intensity exercise enhances early muscle glycogen resynthesis in horses. Exp Physiol.

[96] Pratt SE, Lawrence LM, Warren LK, Powell DM (2005). The effect of exercise on the clearance of infused acetate in the horse. J Equine Vet Sci.

[97] Frampton J, Murphy KG, Frost G, Chambers ES (2020). Short-chain fatty acids as potential regulators of skeletal muscle metabolism and function. Nat Metab.

[98] Huang L, Li T, Zhou M, Deng M, Zhang L, Yi L (2021). Hypoxia improves endurance performance by enhancing short chain fatty acids production via gut microbiota remodeling. Front Microbiol.

[99] Pan JH, Kim JH, Kim HM, Lee ES, Shin DH, Kim S (2015). Acetic acid enhances endurance capacity of exercise-trained mice by increasing skeletal muscle oxidative properties. Biosci Biotechnol Biochem.

