# Supplementary material for: Expanded catalogue of metagenome-assembled genomes reveals resistome characteristics and athletic performance-associated microbes in horse
Source: Microbiome. 2023 Jan 12;11:7. doi: 10.1186/s40168-022-01448-z (PMC9835274; doi:10.1186/s40168-022-01448-z)
Supplement: Supplementary file 5 — Additional file 4: Figure S1. Rarefaction curve of the gene numbers in our samples. The curve nearly plateaus when sufficient sequence data are included, with few novel genes being left undetected. [file 40168_2022_1448_MOESM4_ESM.pdf]

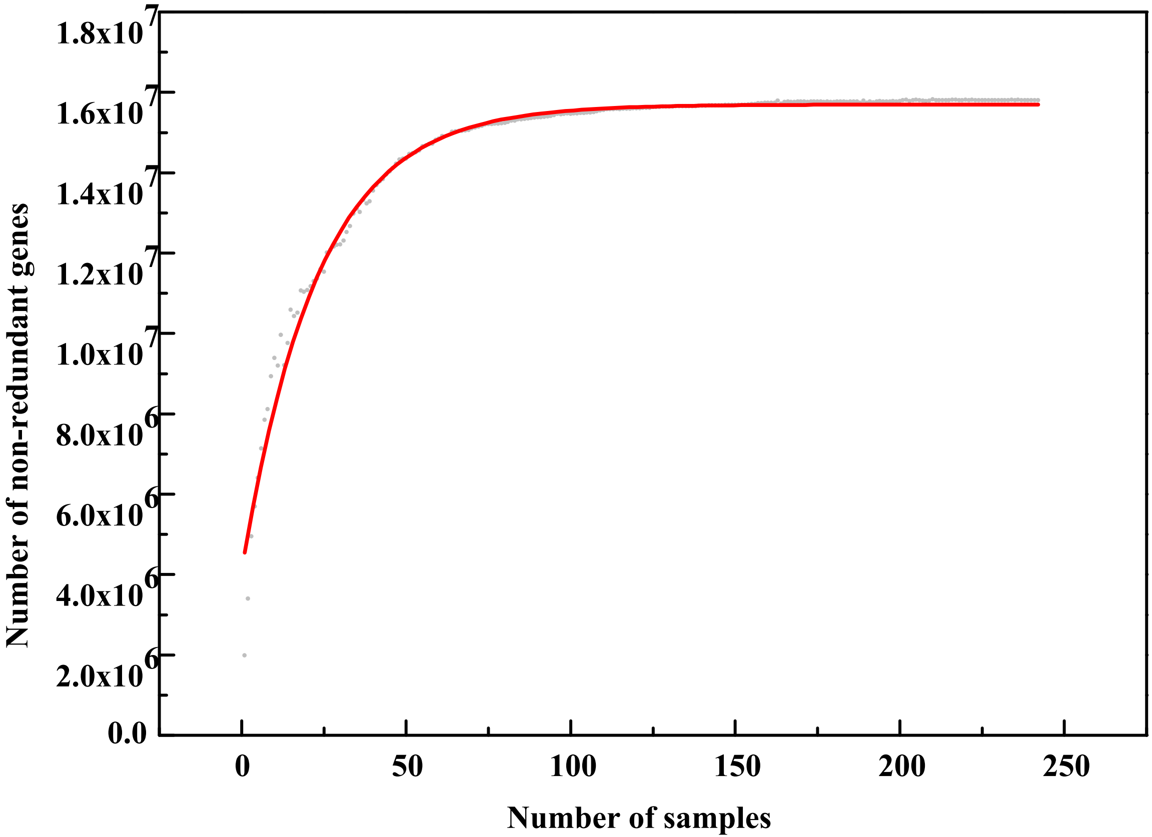

Figure S1. Rarefaction curve of the gene numbers in our samples. The curve nearly plateaus when sufficient sequence data are included, with few novel genes being left undetected.
