# Supplementary material for: Expanded catalogue of metagenome-assembled genomes reveals resistome characteristics and athletic performance-associated microbes in horse
Source: Microbiome. 2023 Jan 12;11:7. doi: 10.1186/s40168-022-01448-z (PMC9835274; doi:10.1186/s40168-022-01448-z)
Supplement: Supplementary file 7 — Additional file 6: Figure S2. Assessment of the degree of contamination and integrity of 2272 high-quality MAG in the horse gut. The grey circles indicate 80–90% genome integrity with 5–10% contamination; the red circles indicate > 90% genome integrity with < 5% contamination. [file 40168_2022_1448_MOESM6_ESM.pdf]

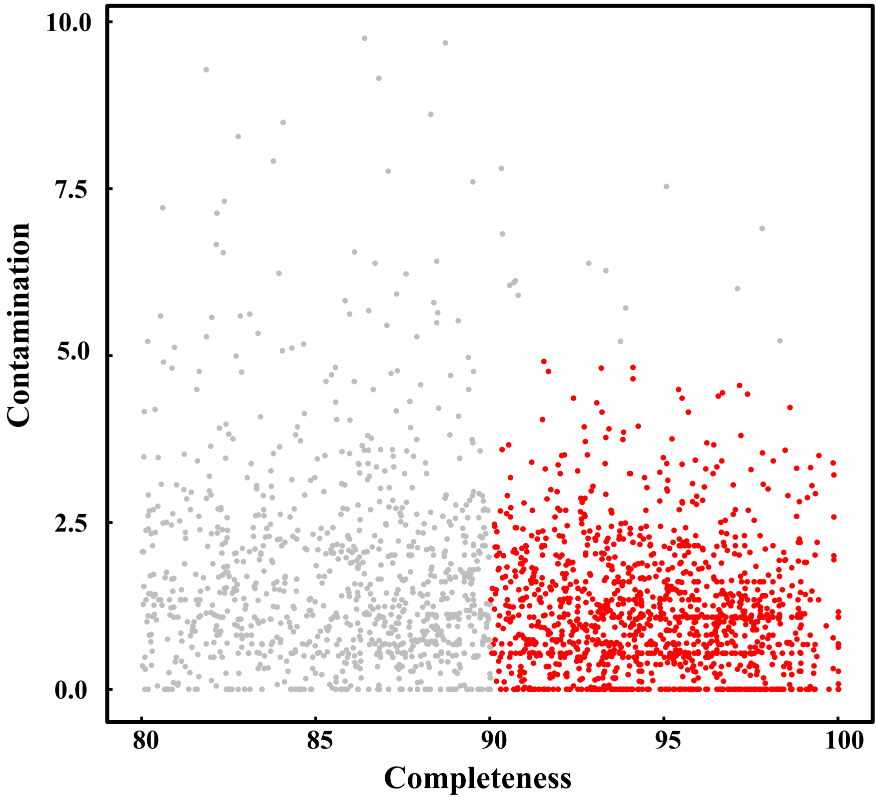

Figure S2. Assessment of the degree of contamination and integrity of 2272 high-quality MAG in the horse gut. The grey circles indicate 80–90% genome integrity with 5–10% contamination; the red circles indicate > 90% genome integrity with < 5% contamination.
