# Supplementary material for: Expanded catalogue of metagenome-assembled genomes reveals resistome characteristics and athletic performance-associated microbes in horse
Source: Microbiome. 2023 Jan 12;11:7. doi: 10.1186/s40168-022-01448-z (PMC9835274; doi:10.1186/s40168-022-01448-z)
Supplement: Supplementary file 14 — Additional file 13: Figure S3. Overview of 11 circular genomes. From the outside to the inside, the concentric circles indicate the positional coordinates of the genomic sequence, coding genes, gene annotation, noncoding RNAs, genomic GC content, and genomic GC skew values. Gene annotation: different colours are used to distinguish different terms of the KEGG and GO annotations. Genome GC content: the inner, red part indicates that the GC content of the region is less than the average GC content of the whole genome; the outer, green part indicates that the GC content of the region is greater than the average value. GC skew value: the inner, pink part indicates that the G content in the area is less than the C content; the outer, light-green part indicates that the G content in the area is greater than the C content. [file 40168_2022_1448_MOESM13_ESM.pdf]

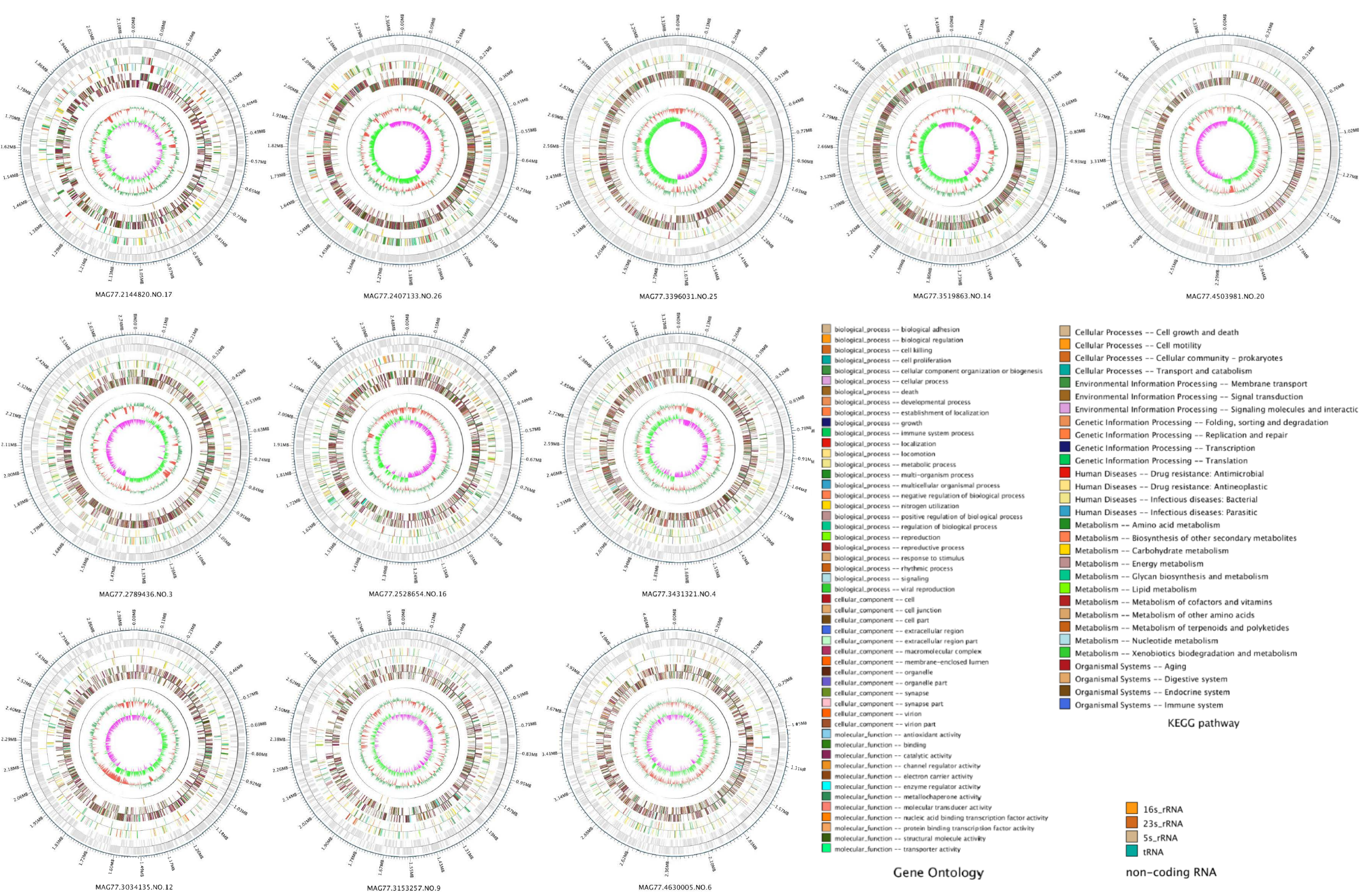

Figure S3. Overview of 11 circular genomes. From the outside to the inside, the concentric circles indicate the positional coordinates of the genomic sequence, coding genes, gene annotation, noncoding RNAs, genomic GC content, and genomic GC skew values. Gene annotation: different colours are used to distinguish different terms of the KEGG and GO annotations. Genome GC content: the inner, red part indicates that the GC content of the region is less than the average GC content of the whole genome; the outer, green part indicates that the GC content of the region is greater than the average value. GC skew value: the inner, pink part indicates that the G content in the area is less than the C content; the outer, light-green part indicates that the G content in the area is greater than the C content.
