# Supplementary material for: Expanded catalogue of metagenome-assembled genomes reveals resistome characteristics and athletic performance-associated microbes in horse
Source: Microbiome. 2023 Jan 12;11:7. doi: 10.1186/s40168-022-01448-z (PMC9835274; doi:10.1186/s40168-022-01448-z)
Supplement: Supplementary file 16 — Additional file 15: Figure S4. Venn plots of the gene catalogues of this study and previous published horse study. Data for published articles are marked with author names. [file 40168_2022_1448_MOESM15_ESM.pdf]

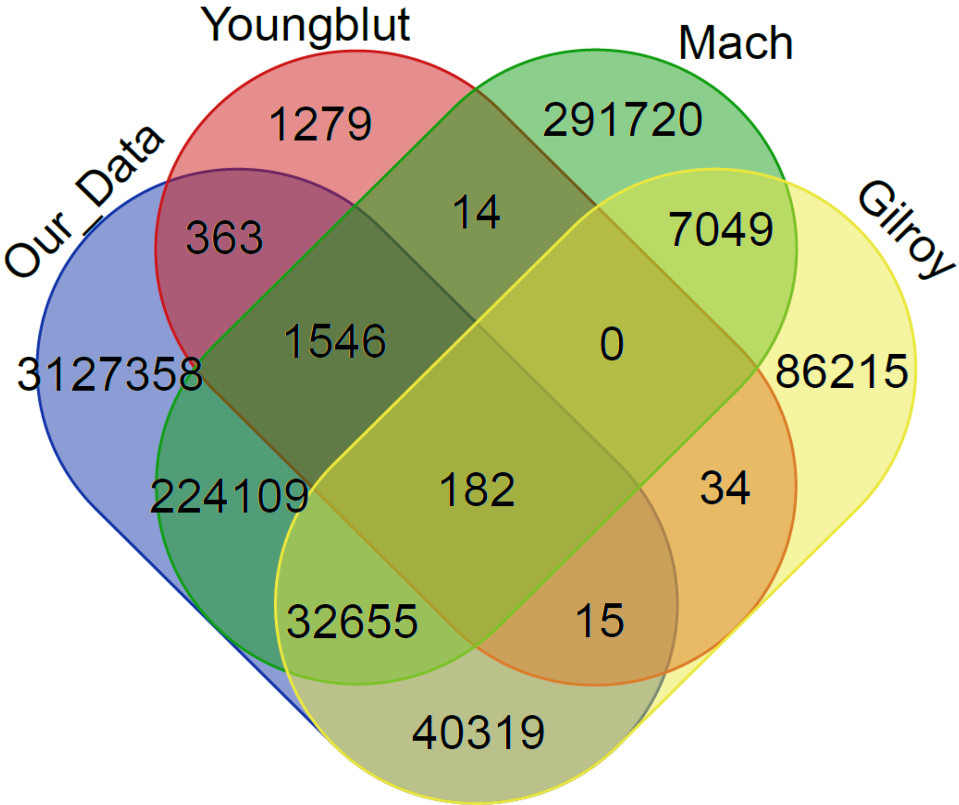

Figure S4. Venn plots of the gene catalogs of this study and previous published horse study. Data for published articles are marked with author names.
