# Supplementary material for: Expanded catalogue of metagenome-assembled genomes reveals resistome characteristics and athletic performance-associated microbes in horse
Source: Microbiome. 2023 Jan 12;11:7. doi: 10.1186/s40168-022-01448-z (PMC9835274; doi:10.1186/s40168-022-01448-z)
Supplement: Supplementary file 22 — Additional file 21: Figure S5. RT–PCR amplification of 10 randomly selected ARG. M is the size marker (Takara DL500: 500 bp, 400 bp, 300 bp, 200 bp, 150 bp, 100 bp, and 50 bp), and N is the negative control. [file 40168_2022_1448_MOESM21_ESM.pdf]

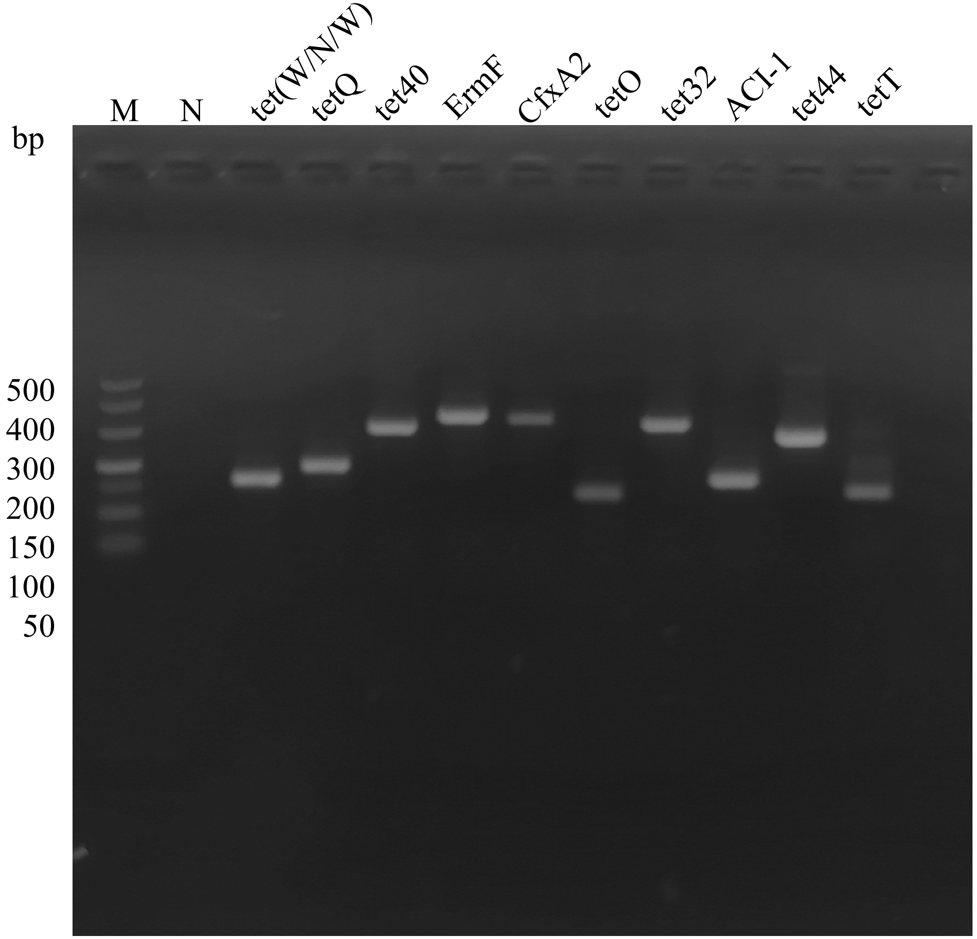

Figure S5. RT–PCR amplification of 10 randomly selected ARG. M is the size marker (Takara DL500: 500 bp, 400 bp, 300 bp, 200 bp, 150 bp, 100 bp, and 50 bp), and N is the negative control.
