# Supplementary material for: Expanded catalogue of metagenome-assembled genomes reveals resistome characteristics and athletic performance-associated microbes in horse
Source: Microbiome. 2023 Jan 12;11:7. doi: 10.1186/s40168-022-01448-z (PMC9835274; doi:10.1186/s40168-022-01448-z)
Supplement: Supplementary file 25 — Additional file 24: Figure S6. The racehorse gut is rich in microbes that can synthesize acetate and butyrate. The microbes enriched in racehorses contain a large number of enzymes that synthesize acetate and butyrate. At the bottom of the heatmap are the difference MAG ID, and the top are the taxonomic information of the MAG. [file 40168_2022_1448_MOESM24_ESM.pdf]

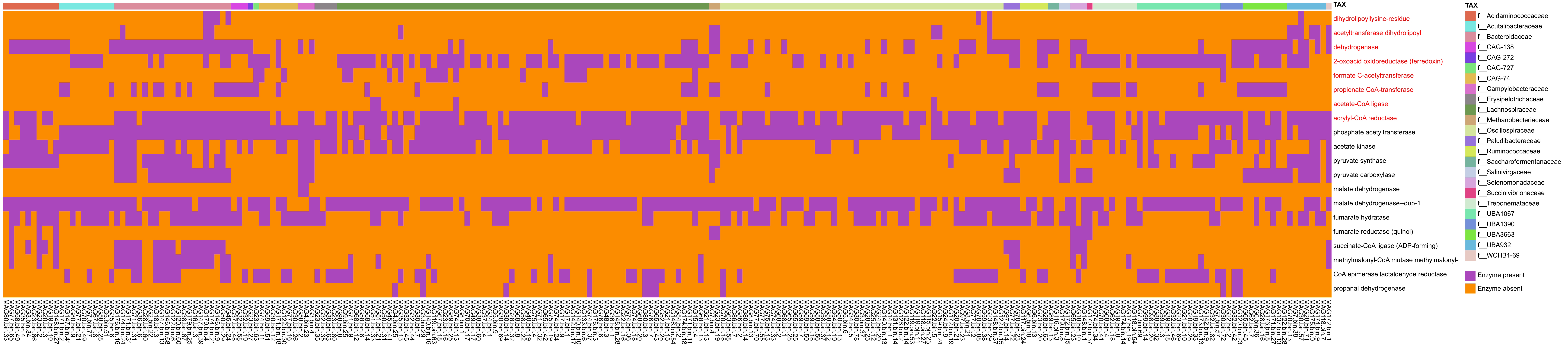

Figure S6. MAG enriched in racehorses contained genes in a major pathway for producing acetate and butyrate. The microbes enriched in racehorses contain a large number of enzymes that synthesize acetate and butyrate. At the bottom of the heatmap are the difference MAG ID, and the top are the taxonomic information of the MAG.
